# The bZIP Proteins of Oncogenic Viruses

**DOI:** 10.3390/v12070757

**Published:** 2020-07-14

**Authors:** Madeleine L. Stolz, Craig McCormick

**Affiliations:** Department of Microbiology & Immunology, Dalhousie University, 5850 College Street, Halifax, NS B3H 4R2, Canada; md412585@dal.ca

**Keywords:** basic leucine zipper (bZIP), herpesvirus, Marek’s disease virus (MDV), Epstein–Barr virus (EBV), Kaposi’s sarcoma-associated herpesvirus (KSHV), human T-cell leukemia virus (HTLV), hepatitis C virus (HCV)

## Abstract

Basic leucine zipper (bZIP) transcription factors (TFs) govern diverse cellular processes and cell fate decisions. The hallmark of the leucine zipper domain is the heptad repeat, with leucine residues at every seventh position in the domain. These leucine residues enable homo- and heterodimerization between ZIP domain α-helices, generating coiled-coil structures that stabilize interactions between adjacent DNA-binding domains and target DNA substrates. Several cancer-causing viruses encode viral bZIP TFs, including human T-cell leukemia virus (HTLV), hepatitis C virus (HCV) and the herpesviruses Marek’s disease virus (MDV), Epstein–Barr virus (EBV) and Kaposi’s sarcoma-associated herpesvirus (KSHV). Here, we provide a comprehensive review of these viral bZIP TFs and their impact on viral replication, host cell responses and cell fate.

## 1. Overview of Basic Leucine Zipper (bZIP) Transcription Factors

**Figure 1:**
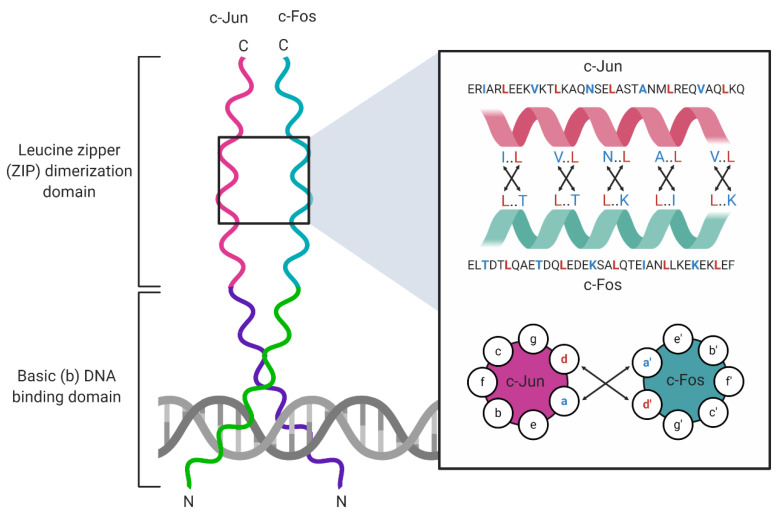
Structure of the c-Jun/c-Fos heterodimer. The c-Jun/c-Fos complexes bind DNA as heterodimers. Each bZIP protein contains a leucine zipper (ZIP) and adjacent basic (b) DNA-binding domain that together constitute the bZIP domain. The ZIP domain organizes into heptad repeats with amino acid residues denoted as positions a–g. Hydrophobic interactions (black arrows) between a (blue) and d (red) residues stabilize dimer formation [6]).

Basic leucine zipper (bZIP) transcription factors (TFs) are eukaryotic DNA-binding proteins that regulate gene expression programs that govern cell proliferation, apoptosis [1,2], response to ER stress, homeostasis [3], and long-term memory [4]. The bZIP TFs contain two common structural motifs: an α-helical leucine zipper (ZIP) dimerization domain, and a DNA-binding domain rich in basic amino acid (aa) residues (Figure 1). The leucine zipper is 60–80 aa in length and organizes into repeats of seven aas (heptad repeats) that contain leucines at every seventh position throughout the domain [5]. Each heptad repeat spans two α-helical turns, allowing ZIP domains to form homodimers or heterodimers with adjacent ZIP α-helices through hydrophobic interactions between complementary leucines and the formation of salt bridges between charged aas. This interaction between two monomers forms a coiled-coil structure [6]. The DNA-binding domain is located adjacent to the ZIP domain [7] and is connected to the first heptad repeat of the leucine zipper by a short hinge region. The DNA-binding domain, also called the basic domain, makes direct contact with specific DNA sequences known as response elements (REs). This domain comprises highly conserved basic amino acid residues that stabilize DNA–protein interactions [5]. The bZIP assembly into homo- and heterodimers controls sequence-specific DNA binding [7,8]. For example, activating transcription factor (ATF) family homodimers recognize 5′-TGACGTCA-3′ cAMP response elements (CREs), and musculoaponeurotic fibrosarcoma (Maf) family homodimers recognize 5′-TGCTGAC(G)TCAGCA-3′ Maf response elements (MAREs), whereas ATF/Maf heterodimers recognize a hybrid 5′-TGCTGACGTCA(C/T)-3′ motif that shares sequence similarities with CREs and MAREs [8]. By contrast, some bZIP heterodimers retain the ability to bind to canonical REs normally bound by each constituent bZIP protein in homodimeric complexes. For example, Jun homodimers bind 5′-TGAG/CTCA-3′ 12-O-tetradecanoylphorbol-13-acetate (TPA) response elements (TREs), and ATF4 homodimers recognize CREs; Jun/ATF4 heterodimers retain the ability to bind both TPA REs (TREs) and CREs [8].

## 2. Cellular bZIP TFs

### 2.1. The AP-1 Complexes

A well-studied example of a heterodimeric bZIP TF complex is the activator-protein 1 (AP-1) complex, which comprises members of the Jun, Fos, ATF, and Maf families of bZIP proteins [1]. Like all TFs, the AP-1 dimer dictates RE binding specificity. For example, Jun/Fos heterodimers recognize the 5′-TGA(G/C)TCA-3′ TRE, whereas ATF homodimers bind the 5′-TGACGTCA-3′ CRE. Jun/Fos heterodimers have been implicated in cell proliferation, apoptosis, and tumorigenesis [1,9]. The best-studied cellular function of the AP-1 complex is cell cycle control, where c-Jun controls the cell cycle by negatively regulating the expression of the tumor suppressor protein p53 and the cyclin-dependent kinase (CDK) inhibitor p21 [10]. The AP-1 complex is regulated at transcriptional and post-translational levels. For example, the transcriptional co-activators yes-associated protein (YAP) and tafazzin (TAZ) induce Fos expression and directly co-operate with the AP-1 complex to facilitate cancer cell proliferation in melanoma [11]. The bZIP broad complex, tramtrack, and bric-à-brac (BTB) domain and cap’n’collar (CNC) homolog 2 (BACH2) controls CD8^+^ T cell differentiation by preventing JunD from accessing specific BACH2 DNA-binding sites that resemble the TPA consensus sequence [12]. Lastly, AP-1 activity is also regulated post-translationally by phosphorylation [13] and O-GlcNAcylation, a post-translational modification that involves the addition of *N*-acetylglucosamine to serine and threonine residues by O-linked glycosylation [14]. Together, these foundational studies of AP-1 complexes have provided keen insights into the structure and function of heterodimeric bZIP complexes. 

### 2.2. The C/EBP Complexes 

The CCAAT/enhancer binding protein (C/EBP) family of bZIPs comprises seven members (C/EBP-α, -β, -γ, -δ, -ε, -ζ, and C/EBP homologous protein (CHOP)) that recognize 5′-(A/G)TTGCG(T/C)AA(T/C)-3′ DNA consensus sites, called CAAT boxes, [15] and facilitate the expression of genes involved in the differentiation of adipocytes [16], myelocytes [17,18], and the trans-differentiation of immortalized murine B cells into macrophages [19]. C/EBPs are also tumor suppressors that regulate the cell cycle by inducing p21 expression [20] and cooperating with p21 to inhibit the cell cycle regulator CDK2 [21]. More recent studies demonstrated the anti-proliferative potential of C/EBPs through their repression of a cluster of microRNAs that induce the expression of the tumor suppressor phosphatase PH domain and leucine rich repeat protein phosphatase 2 (PHLPP2) in acute myeloid leukemia (AML) cells [22]. Conversely, C/EBPs regulate the differentiation of immunosuppressive and tumorigenic myeloid-derived suppressor cells (MDSCs) [23], suggesting a role of C/EBPs in tumorigenesis. 

Early studies demonstrated that C/EBPs can form homodimers or heterodimerize with other C/EBP family members in vitro. A protein microarray comprising 49 human bZIP peptides was employed to demonstrate that C/EBP family members can heterodimerize with ATF family members [24]. Moreover, C/EBPγ and CHOP were shown to specifically interact with individual members of the Fos and Maf family of bZIPs [24]. The formation of C/EBPβ/C/EBPγ heterodimers [25] and C/EBPγ/ATF4 heterodimers [26] was also detected in vitro using electrophoretic mobility shift assays (EMSAs). Furthermore, C/EBPα/c-Jun heterodimers can bind a hybrid TRE and CAAT box REs (5′-TGACGCAAT-3′) with a higher affinity than C/EBP homodimers. C/EBPα/c-Jun and C/EBPα/c-Fos heterodimers also potently drive monocyte differentiation in mouse bone marrow mononuclear cells compared to weaker C/EBPα homodimers and c-Jun/c-Fos heterodimers [27]. Together, these studies of C/EBP complexes have reinforced the idea that heterodimer formation diversifies the transcriptional output of bZIP TFs.

### 2.3. The CREB Complexes

Divergent bZIP proteins can bind common REs. For example, the CREs bound by the aforementioned ATF bZIP proteins are also bound by cAMP response element-binding protein (CREB) family members [4]. Early studies implicated CREBs in long-term memory in the *Aplysia* genus of sea slugs [28], conditioned fear memory in transgenic mice [29], and the survival of sympathetic neurons [30]. The targets of CREBs include neutrophin-encoding genes, the products of which are neuronal growth factors that control cell survival [31] and synaptic function [32]. Like other bZIP TFs, CREBs are subject to extensive regulation by post-translational modifications. For example, the mitogen- and stress-activated kinase 1 (MSK1) [33] and the serine/threonine kinase Akt/protein kinase B (PKB) [34] activate CREB by phosphorylation at Ser133. By contrast, the transducers of regulated CREB activity (TORCs) activate CREB by direct binding to CREB bZIP domains [4,35]. Newman and Keating conducted studies using protein microarrays to identify potential interactions amongst human bZIPs. They reported that CREBs can form heterodimers with members of their own family, as well as with nuclear factor interleukin 3 regulated (NFIL3) [24]. The bZIP NFIL3 represses CREB-inducible genes [36] and C/EBP-inducible genes by competing for binding to common DNA motifs [37]. Heterodimer formation between CREB and NFIL3 was later confirmed in vitro by competitive EMSAs. Conversely, NFIL3 did not form heterodimers with C/EBPα [38]. Thus, the accumulating evidence indicates that, like other bZIP TFs, the transcriptional output of CREB family proteins is strongly influenced by homo- and heterodimerization. 

## 3. The bZIP Transcription Factors of the Unfolded Protein Response

The unfolded protein response (UPR) is a cellular stress response that responds to the accumulation of unfolded proteins in the endoplasmic reticulum (ER), the site of synthesis and folding of secreted and transmembrane proteins [3]. Nutrient deprivation, Ca^2+^ depletion, hypoxia, heart disease, diabetes, and viral infection are all factors that can cause the accumulation of unfolded proteins in the ER lumen and trigger stress responses [39]. Cells sense ER stress through the ER-resident transmembrane receptors inositol-requiring enzyme 1 (IRE1), protein kinase R-like endoplasmic reticulum kinase (PERK), and ATF6. In the absence of ER stress, the lumenal domains of IRE1, PERK, and ATF6 are bound by the cellular chaperone binding immunoglobulin protein (BiP), which represses the activation of each of these sensors [40]. BiP is modified by the adenylyltransferase FIC-domain-containing ER-localized enzyme (FICD)-mediated conjugation of adenosine monophosphate (AMP); this post-translational modification is commonly known as AMPylation. BiP–AMP represses the UPR sensors. When unfolded proteins accumulate in the ER, FICD de-AMPylates BiP, which increases affinity for unfolded substrate proteins [41], allowing it to dissociate from stress sensors and activate the UPR [42]. The IRE1, PERK, and ATF6 arms of the UPR work collectively to increase ER folding capacity and restore homeostasis. This includes mediating the degradation of unfolded proteins, upregulating chaperone synthesis, and increasing lipid biogenesis. If ER stress cannot be resolved, the cell switches from an adaptive response to an apoptotic response [3]. 

The transcription factors X-box binding protein 1 spliced (XBP1s), ATF4, CHOP, and ATF6 (N-terminus) (ATF6(N)) are bZIP TFs that operate downstream from UPR sensors to transactivate UPR genes. Following UPR activation, ATF6 relocates from the ER to the Golgi apparatus and is processed into ATF6(N) by site-1 protease (S1P) and site-2 protease (S2P) [43]. The newly truncated ATF6(N) protein translocates to the nucleus and binds the CRE-like consensus site 5′-TGACGTG(G)-3′ to transactivate BiP and other UPR genes [44]. ATF6(N) also transactivates *Xbp1*, providing additional *Xbp1* mRNAs that can be processed by IRE1. IRE1 directly splices the *Xbp1* transcript to produce transcriptionally active XBP1s [45]. ATF6(N) and XBP1s form heterodimers in vitro [24,38] to induce the transcription of ER-associated degradation (ERAD) genes [46]. The dimerization status and function of ATF6(N) may vary during ER stress. As such, ATF6(N) drives the transcription of chaperone genes as a homodimer and switches to forming ATF6(N) and XBP1s heterodimers later during ER stress to drive ERAD [46]. Further studies on the heterodimerization properties of ATF6(N) and XBP1s have not been performed to date.

PERK phosphorylates and inactivates eukaryotic initiation factor 2α (eIF2α), which downregulates global translation [3]. Some mRNAs, such as the *ATF4* transcript, contain short upstream ORFs in the 5′ untranslated region of the mRNA. The uORFs exert negative effects on downstream translation; uORF translation can cause the dissociation of the eIF2-GTP/Met-tRNAi ternary complex from the mRNA, ribosome dissociation, or stalling [47]. However, during ER stress, uORF-containing transcripts like *ATF4* are preferentially translated [3]. ATF4 is translated via a uORF-skipping mechanism and induces the expression of CHOP, which is involved in the regulation of apoptosis. Concurrently, ATF4 also upregulates the expression of growth arrest and DNA damage-inducible protein 34 (GADD34), which recruits protein phosphatase 1 (PP1) to dephosphorylate eIF2α [3,48]. Jun, Fos, and the C/EBP family of bZIPs are potential binding partners of ATF4 [24]. Indeed, immunoblot analysis revealed the existence of ATF4/C/EBP heterodimers and ATF4/Maf heterodimers in mice *in vivo* [49]. ATF4/C/EBPγ heterodimers bind 5′-ATTGCATCA-3′ C/EBP/ATF response elements (CAREs)—also called amino acid response elements (AAREs)—to transactivate stress-responsive genes as part of the integrated stress response [26]. By contrast, ATF4/CHOP heterodimers inhibit the ATF4-mediated transcription of the asparagine synthetase (*ASNS*) gene from the AARE site in the *ASNS* promoter [50], which indicates that CHOP may also heterodimerize with Jun or Fos and bind TREs as heterodimers to regulate the transcription of collagenase and somatostatin genes from their respective promoters [51]. Potential binding partners of CHOP beyond ATF4, AP-1, or members of the C/EBP family of bZIPs have not been studied to date.

Together, these studies have shaped our understanding of how families of cellular bZIP TFs coordinate transcription. It has become clear that the formation of bZIP homodimers and heterodimers, and the post-translational modifications of these complexes, allow cell signaling events to fine-tune transcriptional output. There is also emerging evidence for competition between bZIP TFs for common DNA-binding sites. Next, we will focus attention on viral bZIP TFs and how they integrate themselves into these cellular networks.

## 4. Viral bZIP Transcription Factors

**Figure 2:**
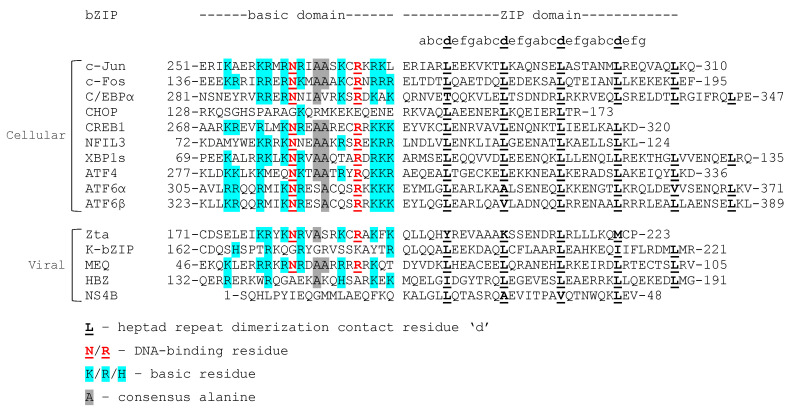
Sequence alignment of viral and human bZIPs. Amino acid sequences were obtained from the National Center for Biotechnology Information (NCBI) gene database and from the UniProt Knowledgebase. Colored residues represent consensus (red and gray) or basic (blue) amino acids. The viral bZIPs Zta, K-bZIP, MEQ, HBZ, and NS4B are expressed by the viruses Epstein-Barr virus, Kaposi’s Sarcoma associated herpesvirus, Marek’s disease virus, human T-lymphotropic virus, and hepatitis C virus, respectively. NCBI Accession Numbers: c-Jun NP_002219.1; c-Fos NP_005243.1; C/EBPα AAC50235.1; CHOP NP_001181982.1; CREB1 NP_001358356.1; NFIL3 NP_001276928.1; XBP1s NP_001073007.1; ATF4 NP_001666.2; ATF6α NP_031374.2; ATF6β NP_004372.3; Zta YP_401673.1; K-bZIP AAD21530.1; MEQ AFU65791.1; HBZ BAX35088.1; NS4B PRO_0000037526.

Through millennia of co-evolution with their hosts, many viruses have acquired host genes that evolve further when mutations that optimize viral fitness are fixed in the genome [52]. Epstein–Barr virus (EBV) and human cytomegalovirus (HCMV), for example, encode interleukin-10 (IL-10) homologs to facilitate immune evasion [53]. Some adeno-, pox-, and herpesviruses encode homologs of the apoptosis regulator B-cell lymphoma 2 (BCL-2) to prevent the receptor-mediated apoptosis of infected cells and promote cell survival [54]. Likewise, some viruses have evolved to encode their own bZIP transcription factors. To date, viral bZIPs have been identified in the human oncoviruses EBV, Kaposi’s sarcoma-associated herpesvirus (KSHV), human T-lymphotropic virus 1 (HTLV-1), and hepatitis C virus (HCV), as well as in the oncogenic chicken herpesvirus Marek’s disease virus (MDV). These five viral bZIPs and their functions will be discussed here in further detail. Viral bZIPs are structurally and functionally similar to cellular bZIPs, but often contain atypical domains (Figure 2). Viral bZIPs can bind cellular counterparts to usurp cellular functions that favor efficient viral replication.

## 5. Zta: The Epstein–Barr Virus (EBV) bZIP Transcription Factor

EBV, also called human gammaherpesvirus 4 (HHV-4), is a human γ-herpesvirus and oncovirus with a large DNA genome of about 170 kilobase pairs (kbp) in size. EBV can be transmitted through saliva [55] and infects tonsillar B cells by membrane fusion at the plasma membrane or in endocytic vesicles [56]. B lymphocytes are the primary reservoir of the virus, but it can also infect epithelial cells. A hallmark of herpesvirus infections is their ability to establish life-long latency in the host. EBV has a latent life cycle, in which only few viral proteins are expressed, and a lytic life cycle characterized by viral replication and virion production. EBV causes infectious mononucleosis, nasopharyngeal carcinoma (NPC), Hodgkin’s lymphoma, Burkitt’s lymphoma, and gastric carcinoma, mainly in immunocompromised individuals, such as AIDS and transplant patients [55]. 

EBV encodes the viral bZIP TF called BamHI Z Epstein–Barr virus replication activator (BZLF1, Zta, ZEBRA) that is expressed during the immediate early phase of lytic replication. Zta orchestrates a variety of functions that include the induction of cell growth arrest [57,58,59] and reactivation from latency [60,61], but is also required for lytic replication [62,63]. Zta recruits the viral helicase BBLF4 to the viral lytic origin of DNA replication (oriLyt), which leads to the assembly of the viral replication complex and genome replication [64]. Zta, in concert with the immediate early replication and transcription activator (Rta) that is likewise required for lytic replication [65], activates the transcription of lytic genes that contain Zta response elements (ZREs) [66].

### 5.1. Zta Structure and Function

Zta is a structurally atypical bZIP protein [67]. Although its carboxy-terminal bZIP dimerization domain is organized into heptad repeats, only one of the repeats contains the characteristic leucine at the seventh aa position (Figure 2). Because of this atypical structure, Zta may not be able to form heterodimers with other bZIPs. Indeed, no heterodimerization partners have been reported to date [68]. The DNA-binding domain of Zta is similar to the DNA-binding domains of cellular and viral bZIPs and contains many basic aa residues, as well as the consensus arginine and asparagine (Figure 2). Zta recognizes and binds to specific 5′-TG(T/A)G(C/T)(A/C)A-3′ ZREs and TREs [67,69,70] and is often referred to as a homolog of Jun and Fos, although the bZIP domain of Zta shares little sequence homology with either (Figure 2). The bZIP domain of Zta, along with the carboxy-terminal (CT) region located adjacent to the bZIP domain, are required for DNA binding and transcriptional activation from ZRE-containing genes [70]. The CT region binds to the bZIP domain through hydrophobic interactions, which increases homodimer stability and enables binding to a wider array of DNA substrates [71,72].

Zta supports lytic viral replication in a variety of ways. For example, Zta interacts with the histone acetylase CREB-binding protein (CBP) [73] and DNA double-stranded break repair protein 53 binding protein 1 (53BP1) in EBV-infected lymphoma cells [74] to transactivate viral genes [73,74]. Zta also binds and recruits mitochondrial single-stranded DNA-binding protein (mtSSB) to the nucleus of EBV-infected lymphoblastoid and lymphoma cells to inhibit mitochondrial DNA replication, which promotes efficient lytic replication by an unknown mechanism [75]. Zta binds ZRE and ZRE-like elements in the promoters of human cytokine genes and drives the transcription of IL-10 [76], IL-8 [77], and IL-13 [78] in infected B cells [76,78] and nasopharyngeal carcinoma cells [77]. This Zta-mediated transactivation of cytokine expression promotes immune evasion and MHC-II downregulation by the inhibition of toll-like receptor (TLR) signaling [76], the expansion of EBV-infected B cell and lymphoblastoid populations [78], and inflammation [77]. Likewise, Zta induces the production of tumor growth factor-β (TGF-β) in HeLa cells and B lymphocyte cell lines [79], as well as TRK-related tyrosine kinase (TKT) in nasopharyngeal carcinoma cells [80], and likely contributes to EBV tumorigenesis [80,81]. 

### 5.2. Zta and Cell Cycle Control

There is strong evidence that Zta regulates the cell cycle to aid viral replication [57,59,82,83]. Early studies showed that Zta activates the p53 tumor suppressor protein and p53-responsive cyclin-dependent kinase (CDK) inhibitors p21 and p27 to enforce G_0_/G_1_ cell growth arrest in HeLa cells and nasopharyngeal epithelial cells [57,59]. The Zta-mediated activation of p21 and p27 can be p53-dependent [57] or p53-independent [59]. Zta-mediated G_0_/G_1_ growth arrest requires the bZIP domain and is ZRE-independent, indicating that Zta affects p53 activity at the post-transcriptional level [59]. Conversely, Zta inhibits p53-mediated transcription in HeLa cells through direct interactions between p53 and the Zta bZIP domain, although p53 is not a bZIP protein [82]. Zta may also inhibit p53 transactivation by reducing the cellular levels of TATA-binding protein (TBP), which is an essential cofactor for p53-mediated transcription [83]. A later study proposed a model for the Zta-mediated modulation of p53 activity, whereby Zta induces p53 activity by enhancing TBP–p53 complex formation early during lytic replication to arrest the cell cycle and create an environment favorable for EBV replication [84]. According to this model, Zta then inhibits p53-mediated transactivation by reducing TBP levels to prevent apoptosis. 

### 5.3. Heterodimer Formation between Zta and Cellular bZIP TFs 

Zta interacts with C/EBPα in EBV-positive cell lines to mediate G_0_/G_1_ cell cycle arrest. Wu and colleagues showed that Zta expression from an adenovirus construct induces C/EBPα and p21 expression *in vitro* and confirmed the interaction between Zta and C/EBPα by co-immunoprecipitation (co-IP) and EMSA. They demonstrated that Zta and C/EBPα co-localize in the nucleus in human primary foreskin fibroblast cells and concluded that Zta and C/EBPα coordinate *p21* transcription [84]. Although the interaction between Zta and C/EBPα is essential for the Zta-mediated p21 induction and requires the bZIP domain of Zta [85], the group later performed a glutaraldehyde cross-linking assay and SDS-PAGE to demonstrate that Zta and C/EBPα do not form heterodimers *in vitro* [86]. 

The Keating lab assessed the potential heterodimer formation between viral and cellular bZIPs using peptide microarrays. They found that Zta readily formed homodimers, but did not form heterodimers with any of the cellular target proteins tested [68], which suggested that the structure of the Zta bZIP domain may favor homodimerization over heterodimerization. The interaction between Zta and C/EBPα may therefore involve the formation of higher-order oligomeric complexes rather than heterodimers [86]. To date, no other studies have investigated heterodimer formation between Zta and cellular bZIPs.

In summary, Zta works in concert with other proteins to bind ZREs and CREs and transactivate viral and host genes, thereby gaining control over cell growth and immune surveillance mechanisms. Zta exclusively forms homodimers but may also form higher-order oligomeric structures that include cellular bZIPs. Strictly speaking, Zta is not an oncogene, but it may initiate the transcription of other viral genes with oncogenic potential. Likewise, Zta induces the transcription of IL-13, a cytokine that drives the proliferation of EBV-infected B cells. Thus, Zta is part of a complex viral transcription program that contributes to oncogenesis. 

## 6. K-bZIP: The Kaposi’s Sarcoma-Associated Herpesvirus (KSHV) bZIP Transcription Factor

KSHV, also called human gammaherpesvirus 8 (HHV-8), is a human oncovirus with a 160–175 kbp genome that primarily infects B lymphocytes, endothelial cells, and epithelial cells [87]. KSHV establishes persistent infection in B lymphocytes and causes Kaposi’s sarcoma (KS), an AIDS-defining cancer, and the lymphoproliferative disorders multicentric Castleman’s disease (MCD) and primary effusion lymphoma (PEL) [87]. As with EBV, immunocompromised individuals are most at risk of developing KSHV-associated disease. Like EBV, KSHV has a latent and lytic life cycle, both of which are linked to oncogenesis [87,88]. 

KSHV encodes the viral bZIP KSHV basic leucine zipper (K-bZIP), also called K8, K8α, or replication-associated protein (RAP), early during lytic viral replication from the *K8* gene. The *K8* pre-mRNA contains four exons and three introns that can be differentially spliced and translated to yield up to four different potential protein isoforms (I-IV), the most abundant of which is the 237 aa K-bZIP [89,90,91]. The relative expression levels of these alternatively spliced isoforms during viral replication remain only partially understood. K-bZIP is a functional homolog of Zta and plays a role as a transactivator and repressor of viral and host genes during lytic KSHV replication. K-bZIP, similarly to Zta, is required for lytic replication and virion production in the KSHV-infected B cell lymphoma cell line BCBL-1 [92,93] and inhibits G1 cell cycle progression to further aid viral replication [94,95].

### 6.1. K-bZIP Structure and DNA Binding 

K-bZIP contains a classical ZIP domain at its carboxy terminus [89]. Apart from one isoleucine, all heptad repeats of the ZIP domain contain leucine at the seventh aa position (Figure 2) [89]. However, the DNA-binding domain of K-bZIP has fewer basic aa residues than other cellular and viral bZIPs and lacks conserved arginine and asparagine residues (Figure 2) [89]. The last thorough review of the herpesvirus-encoded bZIPs Zta and K-bZIP, published in 2003, stated that no direct interaction between K-bZIP and DNA had been reported, perhaps because K-bZIP lacks a classical basic domain [96]. However, homo- and heterodimerization between bZIPs affect DNA binding and sequence specificity [8]. As such, K-bZIP may interact with DNA as a homodimer or in concert with other proteins, viral or cellular, despite its atypical basic domain. Indeed, K-bZIP is required for oriLyt-dependent KSHV replication in African green monkey kidney (Vero) cells transfected with the viral oriLyt region [92]. The KSHV lytic switch protein K-Rta, the KSHV-encoded homolog of EBV Rta, interacts and colocalizes with K-bZIP and other viral proteins in viral replication compartments to assemble a pre-replication complex at the oriLyt to initiate viral DNA replication. K-Rta and K-bZIP may recruit the pre-replication complex to the oriLyt by engaging with their respective DNA-binding sites in the oriLyt region [97]. In this model, K-bZIP binds to a cluster of C/EBP binding motifs [97].

Another study performed by Ellison and colleagues identified K-Rta- and K-bZIP-responsive promoters on the KSHV genome; K-bZIP alone transactivated 21 of 83 KSHV promoters tested. They also performed a ChIP assay in TREx-BCBL-1-Rta cells to confirm that the respective K-Rta- and K-bZIP-responsive promoters are indeed occupied by the two proteins. Interestingly, the co-expression of K-Rta and K-bZIP revealed that K-bZIP modulates the K-Rta-mediated transactivation of viral promoters, repressing expression from K-Rta-responsive early lytic promoters, including the K8 promoter, but enhancing expression from all other K-Rta-responsive lytic promoters [98]. Thus, K-bZIP is required to precisely regulate viral gene expression. 

### 6.2. K-bZIP Function and Role as a Transactivator and Repressor 

K-bZIP, similarly to Zta, co-operates with the immediate early K-Rta protein to regulate viral gene expression during lytic replication [98,99,100]. Two independent studies investigated possible interactions between K-bZIP and K-Rta and showed by immunostaining that the two proteins co-localize in the nucleus of lytic BCBL-1 cells [99], as well as transfected HeLa cells [100]. These studies also demonstrated that K-bZIP and K-Rta associate *in vitro* via co-immunoprecipitation experiments. The interaction between the two proteins requires a region in the carboxy-terminus of K-bZIP, the identity of which is in question [99,100]. The DNA-binding domain and amino-terminus of K-bZIP are required to associate with K-Rta in BCBL-1 cells [99], whereas the ZIP domain is required for the association with K-Rta and the repression of K-Rta-mediated transactivation in 293T cells [100]. To date, no further studies have investigated the nature of the protein–protein interaction between the two proteins. K-bZIP modulates the transactivation activity of K-Rta to shift viral gene expression away from the expression of immediate early genes [98]. K-bZIP and K-Rta can also associate with cellular promoters to transactivate host gene expression [101]. A recent study identified a novel 5′-AAAATGAAAA-3′ K-bZIP-binding motif in viral and cellular promoters by ChIP-seq in TREx-BCBL-1-Rta cells [101]. The viral genes containing this motif include all classes of lytic genes (early and late), as well as the *K8* gene. The cellular genes containing the motif encode collagen type IV alpha-3-binding protein (COL4a3BP), an ER transmembrane transporter of ceramide; deleted in malignant brain tumors 1 protein (DMBT1), a candidate tumor suppressor; melanoma-associated antigen C3 (MAGEC3), a tumor antigen; ubiquitin-protein ligase E3A (UBE3A), a ubiquitin ligase of the proteasomal pathway; cell division cycle 7-related protein kinase (CDC7), a cell cycle regulating kinase; and Rho associated coiled-coil-containing protein kinase 1 pseudogene 1 (ROCK1P1), a pseudogene. K-bZIP can induce transcription from luciferase constructs containing the novel motif, but how K-bZIP affects de novo transcription from the above-mentioned genes requires further investigation. It is also not yet known whether other viral bZIP proteins bind this motif.

### 6.3. K-bZIP, the Cell Cycle, and Interaction with C/EBPα 

**Figure 3:**
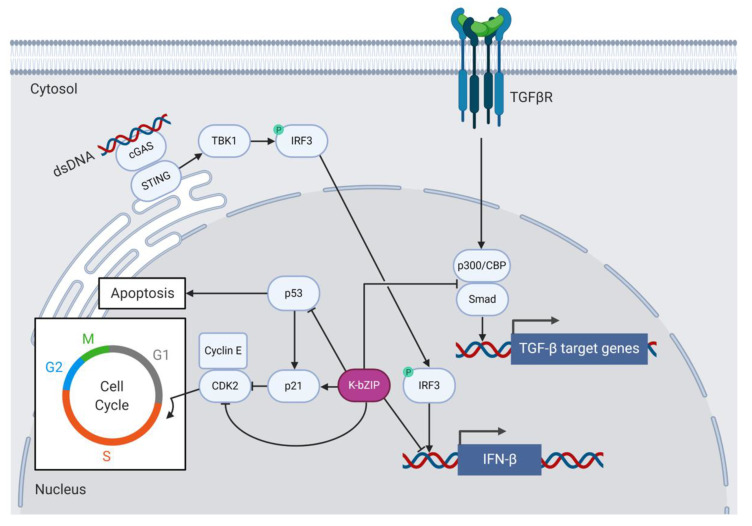
K-bZIP inhibits antiviral innate immune signaling and anti-proliferative signaling. Viral cytosolic dsDNA activates the cGAS/STING pathway, which recruits TBK1 to phosphorylate and activate IRF3. IRF3 transitions to the nucleus to turn on expression of antiviral type 1 IFN genes. The early lytic viral transcription factor K-bZIP prevents IRF3 promoter access to inhibit IFN-β production. K-bZIP inhibits p53 and CDK2 but activates p21 to cause G_0_/G_1_ cell cycle arrest and prevent apoptosis. K-bZIP also prevents the association of p300/CBP with Smad proteins to inhibit expression of TGF-β-responsive genes in response to TGF-β signaling.

Like Zta, K-bZIP regulates the cell cycle and causes growth arrest to aid viral replication [95,102] (Figure 3). K-bZIP delays G_0_/G_1_ growth phase progression by binding and inhibiting cyclin-dependent kinase 2 (CDK2) in BCBL-1 cells. The interaction between K-bZIP and CDK2 requires the basic domain of K-bZIP [95]. K-bZIP expression also induces C/EBPα and p21 in HeLa cells and stalls G_1_ to S phase progression in human diploid fibroblast cells [102]. K-bZIP also associates with C/EBPα and p21 in vitro and modulates their activity to affect cell cycle regulation at the post-transcriptional level [94]. K-bZIP, like Zta, binds C/EBPα and this interaction requires the ZIP domain of K-bZIP. Glutaraldehyde cross-linking and SDS-PAGE experiments demonstrated that K-bZIP and C/EBPα do not form heterodimers [94], suggesting that they may instead associate via higher-order oligomers. The more recent peptide microarray experiments that the Keating lab conducted to detect bZIP heterodimers confirmed the lack of K-bZIP heterodimer formation for all bZIP TFs tested, with the possible exception of ATF2 and ATF7 [68]. Heterodimer formation between K-bZIP and ATF2/7 or other cellular bZIPs has not been further investigated to date. K-bZIP does, however, from strong homodimers [68].

Type 1 interferons (IFNs) are produced in response to viral infection and constitute signaling pathways that induce a range of cellular processes involved in antigen presentation, the induction of apoptosis and the inhibition of viral replication and gene expression [103]. Many viruses, including KSHV, have evolved to subvert host cell innate immune responses to facilitate immune evasion and promote ongoing viral replication. For example, K-bZIP assists KSHV by dampening host antiviral type 1 IFN responses [91,98] (Figure 3). K-bZIP associates with the IFN-β promoter and prevents interferon response factor 3 (IRF3) from binding and activating IFN-β transcription in K-bZIP-expressing 293T cells [98]. Downstream of type 1 IFN expression, K-bZIP interferes with the expression of the IFN-α-responsive genes encoding the antiviral 2′-5′ oligoadenylate synthetase (2′-5′ OAS) and the cytokine interferon-stimulated gene 15 (ISG15) in K-bZIP-expressing 293T cells [91].

The cellular tumor suppressor proteins p53 and promyelocytic leukemia protein (PML) are also activated in response to type 1 IFN signaling and represent pathways to counter viral infection by inducing cellular senescence and apoptosis [104]. The *PML* gene can be activated by IFN-α, IFN-β, and IFN-γ in response to viral infection or in cancer cells [105] and leads to the acetylation, and therefore activation, of p53 and cell cycle arrest [106]. K-bZIP binds and inhibits p53 downstream of IFN signaling, thereby preventing the apoptosis of KSHV-infected cells [107]. An early study showed that co-transfection of a p53-deficient cervical cancer cell line (C33A) with K-bZIP and p53 inhibited gene expression from a p53-dependent luciferase reporter gene. The ZIP domain of K-bZIP binds to p53 [107], as was observed for Zta [82]. A later study also found that K-bZIP recruits p53 to promyelocytic leukemia protein (PML) bodies in PEL cells [108]. PML-deficient PEL cells displayed reduced viral DNA replication, late gene expression, and virion production, which suggests that PML is important to the viral lytic life cycle. Furthermore, K-bZIP was one of several viral proteins shown to interact with PML [109]. The functional relevance of the localization of p53 to PML bodies during lytic KSHV infection remains to be determined. 

### 6.4. K-bZIP Function and Protein–Protein Interactions Not Shared with Zta 

K-bZIP, like Zta, associates with the histone acetylase CBP in vitro, but unlike Zta, K-bZIP does not associate with CBP to drive transcription from lytic KSHV promoters [73,110]. Instead, K-bZIP competes with Smad proteins for CBP and therefore prevents the Smad/CBP association to Smad-responsive promoters downstream of TGF-β signaling in human reporter cell lines (Figure 3). The K-bZIP-mediated inhibition of TGF-β signaling may contribute to KSHV tumorigenesis [110]. K-bZIP also inhibits the function of CBP as a transcription co-factor and represses CBP-dependent gene expression from a TRE-containing luciferase construct in KSHV/EBV-negative follicular B cell lymphoma (BJAB) cells [111].

K-bZIP co-localizes with histone deacetylase (HDAC) proteins in PML bodies and reduces their activity. However, K-bZIP–HDAC complexes also inhibit transcriptional activation from K-Rta and oriLyt promoters, which regulates viral gene expression and replication [112]. K-bZIP interacts with the chromatin remodeling complex subunit SNF5 in yeast to drive transcription. K-bZIP also binds hSNF5, the human homolog of SNF5, in BCBL-1 cells, but does not require hSNF5 to activate genes in mammalian systems. Thus, the accumulating evidence suggests that K-bZIP binds to multiple tumor suppressor proteins like SNF5, which may contribute to viral oncogenesis [113].

Lastly, K-bZIP associates with the viral proteins ORF57 [114] and viral protein kinase (vPK) [115] in TREx-BCBL-Rta cells [114,115]. K-bZIP is phosphorylated by vPK at threonine 111, which relieves the K-bZIP-mediated repression of K-Rta. The K-bZIP/vPK interaction and subsequent K-bZIP phosphorylation may switch K-bZIP from functioning as a repressor of immediate early gene expression to a transactivator of early and late gene expression [115]. Furthermore, the interactions between K-bZIP and K-Rta [100] or K-bZIP and ORF57 [114] may establish a feedback mechanism by which these proteins regulate transcription from their own promoters [100,114]. These viral protein–protein interactions may serve to modulate the respective functions of K-bZIP in a timely manner and may represent a mechanism to fine-tune lytic KSHV replication [114,115]. For example, early during lytic replication, the role of K-bZIP may be to inhibit the K-Rta-mediated expression of early viral genes, such as ORF57 and K8, to assist the transition to late gene expression. Then, later during lytic replication, K-bZIP could assist K-Rta in the expression of viral structural proteins and other late gene products.

### 6.5. The Role of SUMOylation during KSHV Lytic Replication 

In addition to its classical, carboxy-terminal bZIP domain, K-bZIP also has an amino-terminal small ubiquitin-like modifier (SUMO) interaction motif (SIM). The SIM confers SUMO E3 ligase activity to K-bZIP and allows the protein to bind and covalently attach SUMO-2 and SUMO-3, two of the four different SUMO isoforms, to single aa residues on itself and other viral and cellular proteins [116]. SUMOylation is a reversible post-translational modification that can alter the function of a protein by affecting its ability to bind proteins or DNA or by changing its intracellular location. SUMOylation controls diverse cellular processes including transcription, DNA replication, DNA damage repair, and cell division. Because many cellular events are regulated by SUMOylation, some viruses, such as human immunodeficiency virus 1 (HIV-1), influenza A virus (IAV), EBV, and KSHV, have evolved mechanisms to manipulate the SUMOylation machinery to aid viral replication and virion production [117]. Many cellular proteins, including the bZIPs Jun/Fos [118], C/EBPβ [119], ATF6 [120], and XBP1s [121], are SUMOylated. SUMOylation negatively or positively affects the transcriptional activity of SUMOylated TFs to provide another layer of transcriptional regulation [122]. How SUMOylation affects bZIP dimer formation and stability remains to be determined.

In the context of KSHV infection, SUMOylation is important for the assembly and disassembly of PML bodies, modulating type 1 IFN responses, and chromatin remodeling during the latent and lytic phases of KSHV infection. At least two viral proteins, K-bZIP and the latency-associated nuclear antigen (LANA), can be SUMOylated [123]. K-bZIP SUMOylation is required for the repression of IFN-α signaling [91] and the repression of the K-Rta transactivation of the ORF57 promoter [90]. K-bZIP-mediated SUMOylation enhances p53 transcriptional activity, and K-bZIP recruitment to p53 target genes is SIM-dependent, which suggests that a SUMO-rich environment stabilizes K-bZIP/p53 interactions [116]. 

SUMOylation also plays an important role in the regulation of lytic gene expression and virion production. Yang and colleagues showed by ChIP and ChIP-seq that, during viral reactivation in dox-inducible BCBL-1 cells, the viral chromatin is enriched with SUMOylated proteins. They transfected a KS cell line with an E3 ligase-deficient mutant of K-bZIP, containing a leucine 75 to alanine aa substitution in the SIM that abrogates SUMO binding, and showed that SUMO enrichment of the viral chromatin is dependent on K-bZIP E3 ubiquitin ligase activity. In their study, the K-bZIP-mediated SUMOylation of chromatin-associated proteins diminished lytic gene expression and virion production. This suggests that K-bZIP-mediated SUMOylation regulates viral lytic gene expression, which may facilitate immune evasion and efficient viral replication [124]. 

Lastly, SUMOylation can serve as a signal for ubiquitination and proteasomal degradation, and therefore affects the half-life of SUMOylated proteins. SUMO-targeting ubiquitin ligases (STUbLs) target SUMOylated proteins for degradation [125]. The immediate early K-Rta protein is a SIM-containing viral STUbL that exhibits SUMO2/3 binding and ubiquitinates proteins conjugated with SUMO2/3 [126]. Because K-bZIP is a viral E3 SUMO ligase that displays SUMO2/3 specificity, and because SUMO2/3 are the preferred substrates for K-Rta-mediated ubiquitination and subsequent proteasomal degradation, K-bZIP and K-Rta may work in concert to regulate cellular protein levels during lytic replication to create an optimal environment for KSHV replication. However, the cellular and viral targets of K-bZIP-mediated SUMOylation are not known, nor is it known the extent to which K-bZIP and K-Rta work in concert to degrade SUMOylated target proteins. In future studies, affinity purification and mass spectrometry approaches could be employed to identify cellular and viral proteins bound by K-bZIP and whether they are SUMOylated. Hits from this unbiased approach could be corroborated by bioinformatic analysis (i.e., SIM identification) and in vitro SUMOylation assays, such as the one proposed by Yang and colleagues [127]. 

In summary, K-bZIP is a viral bZIP and SUMO E3 ligase expressed early during lytic KSHV replication. Despite an atypical basic domain, K-bZIP binds viral and cellular gene promoters as a homodimer or in concert with other transcription factors, including the viral immediate early K-Rta to assist with viral DNA replication, lytic gene expression, cell cycle arrest, and immune evasion. There is no evidence that K-bZIP is an oncogene, but it contributes to oncogenesis indirectly. Like Zta, K-bZIP exclusively forms homodimers, but may associate with other bZIPs as higher-order oligomers. The role of K-bZIP as a SUMO E3 ligase during lytic viral replication remains poorly understood and warrants further study.

## 7. MEQ: The Marek’s Disease Virus (MDV) bZIP Transcription Factor 

MDV is an oncogenic alphaherpesvirus that infects chickens. Infected birds shed virus from their wings, and transmission occurs when healthy birds inhale the feather dander, also called feather dust, from infected birds [128]. In the lungs, MDV infects macrophages that carry the virus to lymphoid tissues, such as the bursa of Fabricus—the site of hematopoiesis in birds—and the spleen, where the virus infects B and T lymphocytes [129]. MDV causes immunosuppression, blindness, and lymphoid tumors in infected birds and therefore poses a threat to the poultry industry [128]. MDV can be prevented with commercially available vaccines, but vaccine efficacy varies between the different MDV serotypes and vaccine breaks are common [129]. Like all herpesviruses, MDV has a latent and lytic life cycle and establishes life-long infection in its host [128,129].

MDV encodes the viral bZIP MDV Eco Q (MEQ) [130] during the latent and lytic life cycles from two copies of the *meq* gene, one located in the 3′ long terminal repeat region (LTR) and the other in the 5′ long internal repeat region (IRL) of the MDV genome [131]. MEQ, like K-bZIP, is subject to alternative splicing. The two major protein products that result from *meq* splicing are the 339 aa MEQ and a shorter MEQ/vIL-8 hybrid protein that contains a truncated bZIP domain [132]. Jarosinski and Schat used the 3′ rapid amplification of cDNA ends (3′ RACE) in MDV-infected chicken lymphoblastoid MSB-1 cells and chick kidney cells harvested from infected birds to identify multiple additional splice variants of MEQ [133]. However, the expression levels of the alternatively spliced isoforms and their function remain unknown. MEQ is expressed in infected CD4^+^ T cells [131,134] and MDV-associated tumors [134] and is an important player in MDV oncogenesis [134,135,136,137,138], virulence [136,139], and immunosuppression [140].

### 7.1. MEQ Structure and Function 

**Figure 4:**
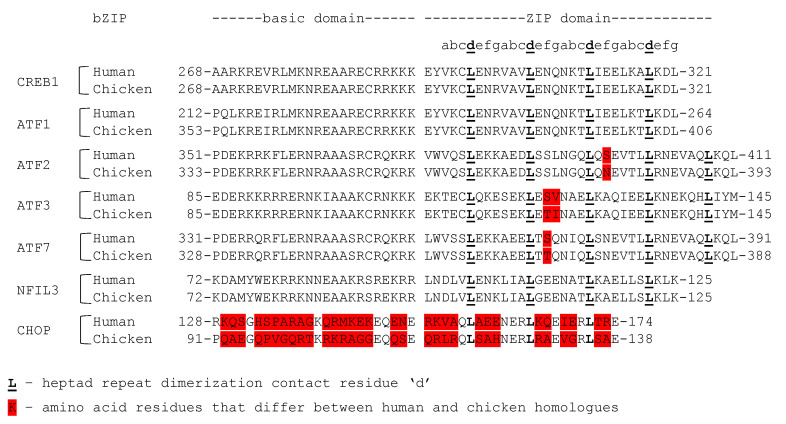
Amino acid similarity of human and chicken bZIP proteins. Amino acid sequences were obtained from the National Center for Biotechnology Information gene database. CREB1 NP_001358356.1 (H) NP_989781.1 (C); ATF1 NP_005162.1 (H) XP_004949774.1 (C); ATF2 NP_001243019.1 (H) NP_990235.1 (C); ATF3 NP_001025458.1 (H) XP_015139364.1 (C); ATF7 NP_001353485.1 (H) XP_025001216.1 (C); NFIL3 NP_001276928.1 (H) NP_989949.1 (C); CHOP NP_001181982.1 (H) XP_015128659.1 (C).

MEQ contains an N-terminal bZIP domain with a classical ZIP domain that resembles c-Jun in length [130] and a basic domain that contains multiple basic residues, as well as the consensus asparagine and arginine (Figure 2). Unlike Zta and K-bZIP, which have only been reported to form homodimers, MEQ can form homodimers and heterodimers [68]. For instance, MEQ heterodimerizes with chicken c-Jun and mouse JunB in vitro and co-localizes with c-Jun in the chicken lymphoblastoid MSB-1 cell line. Glutathione S-transferase (GST) pulldown assays also showed that MEQ heterodimerizes with CREB, ATF1, ATF2, and ATF3 [141]. The Keating lab used peptide microarrays to identify bZIP–bZIP interactions, and detected heterodimer formation between MEQ and the human Jun proteins, ATF1, ATF2, ATF3, and CREB1 (in agreement with the Levy study) and also detected heterodimer formation between MEQ and ATF7, NFIL3, and CHOP [68]. The bZIP domains are identical or nearly identical between the chicken and human homologs of all the above-mentioned proteins, except for CHOP (Figure 4). Cross-species interactions between MEQ and the human bZIPs, assessed by the Keating lab, are possible and might reflect the interactions between MEQ and the chicken homologs of these proteins. The Keating lab did not test dimer formation between full-length proteins, but only their respective bZIP domains. As such, the effect of any possible aa difference between chicken and human homologs outside of the bZIP domain on dimer formation were not assessed. MEQ and Jun heterodimers bind TREs and CREs to transactivate expression from a reporter plasmid containing the *meq* promoter in chicken embryo fibroblasts (CEF cells) [142] and activate gene expression from a *meq* reporter luciferase plasmid more strongly than MEQ homodimers do [141]. MEQ homodimers display less binding specificity for TREs and CREs than MEQ heterodimers, but recognize a unique 5′-RACACACAY-3′ motif, where R represents a purine and Y represents a pyrimidine [143]. The 5′-RACACACAY-3′ motif is found in the viral origin of replication [141]. Whereas MEQ and Jun heterodimers activate transcription, MEQ homodimers repress transactivation from a dual-luciferase construct containing the bidirectional pp14 and pp38 promoters flanking the origin of replication in chicken fibroblast DF-1 cells [141]. As such, MEQ homodimers act as transcriptional repressors, whereas MEQ heterodimers act as transcriptional activators [141].

Besides an N-terminal bZIP domain, MEQ contains a C-terminal proline-rich domain that, when fused to the Gal4 DNA-binding domain, has transactivating and repressive functions that remain poorly understood [142]. The proline-rich repeats of MEQ may also contribute to virulence, because MDV strains that vary in virulence contain polymorphisms in the proline-rich region of the *meq* gene, among other genes [139]. However, the function of the proline-rich domain and its involvement in MDV virulence remain unknown.

### 7.2. MEQ is an Oncogene 

The role of MEQ in MDV-driven oncogenesis has been extensively studied. For instance, the ectopic expression of MEQ in Rat-2 fibroblasts [134] and DF-1 cells [137] induces cell transformation. The infection of chickens with a recombinant *meq* knock-out mutant virus results in no MDV-associated mortality, whereas infection with a reconstituted, MEQ-encoding virus causes significant mortality, the formation of lesions, and the atrophy of lymphoid organs [136]. Transformation-related genes expressed through the v-Jun signaling cascade are upregulated in DF-1 cells transfected with MEQ. RNA silencing of MEQ or Jun in MEQ-expressing DF-1 cells abrogates the upregulation of v-Jun-responsive genes. Thus, MEQ and Jun may work in concert to induce the transformation of DF-1 cells via the v-Jun pathway [137]. MEQ dimerization is essential for MDV-associated oncogenesis [138,144,145]. Inbred, pathogen-free chickens and outbred chickens infected with homo- and dimerization-defective MEQ mutant viruses for 90 days survive infection with the dimerization-defective MEQ mutant viruses, whereas all inbred chickens succumb to the wild type virus within 70 days and 45% of outbred chickens succumb to wild type virus within 90 days. As such, MEQ homo- and heterodimers are essential for MDV-associated oncogenesis [138]. These findings are in line with conclusions from two studies by Suchodolski and colleagues that investigated the ability of a homodimerization-restricted MEQ mutant containing the bZIP domain of the yeast GCN4 [144] and heterodimerization-restricted MEQ mutant containing the bZIP domain of Fos [145] to induce oncogenesis and cell transformation in specific-pathogen-free (SPF) chickens. The association of MEQ with C-terminal binding protein (CtBP) in DF-1 cells [146] and heat-shock protein 70 (Hsp70) in MSB-1 cells [147] via protein–protein interactions that do not involve dimer formation may also be involved in MDV-associated oncogenesis. However, the precise mechanisms by which MEQ induces oncogenesis as homodimers or MEQ and Jun heterodimers, or in concert with other proteins, remain unclear.

MEQ supports MDV replication and promotes oncogenesis by facilitating immune evasion and immunosuppression in infected chickens [140,148]. Li and interferon response factor 7 (IRF7) chickens lack IRF3 and instead deploy IRF7 to drive IFN signaling in vitro. The association of MEQ with STING and IFN7 prevents the assembly of the STING-TANK-binding kinase 1 (TBK1)–IRF7 complex in DF-1 cells and inhibits IFN-β production during the late phase of the lytic cycle [148].

MEQ also assists in MDV-associated oncogenesis by inhibiting the apoptosis of infected cells [134,149,150]. For example, MEQ binds and inhibits the apoptosis inducer apoptin in DF-1 cells [149]. Likewise, MEQ also binds p53 and inhibits its ability to transactivate expression from a luciferase reporter plasmid in MEQ-transfected human non-small lung cancer H1299 cells and prevents p53-mediated apoptosis in CEF cells [150]. MEQ also inhibits apoptosis via the phosphatidylinositol 3-kinase (PI3K)/Akt pathway. PI3K phosphorylates and activates Akt, which then initiates a signaling cascade that results in the expression of genes involved in cell growth and survival. MEQ binds the p85 subunit of PI3K *in vitro* to induce PI3K-dependent Akt phosphorylation in DF-1 cells and promotes survival and viral replication during early MDV infection of CEF cells [151]. The interactions between MEQ and apoptin, p53, or the p85 subunit of PI3K do not involve dimer formation [149,150,151]. Lastly, ectopic MEQ expression upregulates expression of the apoptosis regulator Bcl-2, whereas it downregulates expression of the apoptosis regulator Bax in Rat-2 cells [134].

Altogether, by readily forming heterodimers with cellular bZIPs, MEQ expands DNA recognition from a unique 5′-RACACACAY-3′ motif to TREs and CREs and shifts from the role of a transcriptional repressor to that of an activator, which is key to its immunomodulatory and anti-apoptotic functions. Further study of these properties of MEQ could advance the understanding of the mechanisms of oncogenesis and the design of new vaccine candidates. 

## 8. HBZ: A Human T-Cell Leukemia Virus (HTLV) bZIP Transcription Factor

HTLV-1 is a human delta-retrovirus with a small, single-stranded positive-sense RNA genome [152]. Because of its small genome size, HTLV-1 employs polycistronic translation, alternative splicing, frameshifting, and the proteolytic cleavage of precursor proteins to maximize the production of protein products with different functions [152]. HTLV-1 is transmitted through bodily fluids and primarily infects CD4^+^ T cells, but also CD8^+^ T cells, B cells, myeloid cells, epithelial cells, and fibroblasts [152]. HTLV-1 enters target cells by receptor-mediated membrane fusion at the plasma membrane following the binding of its envelope glycoprotein to the cellular glucose transporter 1 (GLUT1), heparin sulfate proteoglycan (HSPG), and VEGF-165 receptor neuropilin-1 (NRP-1) receptor complex [152]. Like the herpesviruses addressed previously, the retrovirus HTLV-1 also has a latent life cycle that is characterized by viral persistence [152]. Herpesviruses maintain their genome in the form of a plasmid-like extrachromosomal element, the episome, that is tethered to the host chromatin by viral proteins [153]. By contrast, HTLV-1 uses its virally encoded reverse transcriptase to convert its RNA genome into double-stranded DNA, which is then incorporated into the host cell genome by the viral integrase [152]. HTLV-1 establishes asymptomatic infection in most individuals, but rare outcomes include adult T-cell leukemia (ATL) and the neurological disease HTLV-associated myelopathy (HAM)/tropical spastic paraparesis (TSP) [152].

The four human T-lymphotropic viruses share significant sequence homology, but only HTLV-1 encodes a bZIP, the HTLV-1 bZIP factor (HBZ) [152,154]. HBZ is encoded from the *hbz* gene located near the 5′ end of the proviral minus (antisense) strand [154]. HBZ is widely expressed in asymptomatic infections, as well as ATL [155] and HAM/TSP [156,157]. The *hbz* mRNA can be alternatively spliced to produce at least two isoforms, spliced HBZ (sHBZ) and unspliced HBZ (usHBZ) [158]. The functions of usHBZ remain poorly understood. During HTLV-1 infection, HBZ represses viral transcription mediated by the potent viral transactivator Tax [159,160], supports HTLV-1 associated oncogenesis [161,162,163,164,165], and prevents the apoptosis of infected cells [166,167]. Unlike Zta, K-bZIP, and MEQ, which are localized in the nuclei of infected cells, HBZ is a shuttling protein that displays altered cellular localization in different HTLV-1-infected cells. HBZ is localized in the nuclei of the peripheral blood mononuclear cells (PBMCs) of ATL patients [156] and in the cytoplasm of PBMCs of asymptomatic carriers [157] and HAM/TSP patients [156,157]. The cytoplasmic localization of HBZ is a marker for HAM/TSP, but the implications of the subcellular localization in the disease states of HTLV-1 infection are not well understood. 

### 8.1. HBZ Structure and Dimerization Partners

HBZ contains a classical C-terminal ZIP domain that is similar in length to those of K-bZIP and MEQ. Like K-bZIP, all heptad repeats of the HBZ ZIP domain, apart from one isoleucine, contain leucine at the seventh aa position. However, HBZ shares no sequence similarity with K-bZIP and MEQ. The basic domain of HBZ contains few of the basic aa residues present in other viral and cellular bZIPs and lacks the consensus asparagine and arginine (Figure 2). The binding of HBZ to DNA has not been observed to date [68]. Monomeric bZIPs cannot associate with DNA on their own [7,8], which may explain why HBZ, which cannot form homodimers, fails to bind to DNA. However, HBZ can recognize and bind CREs [163] and 5′-TGCTGAC(G)TCAGCA-3′ MAREs [168] when incorporated into heterodimeric bZIP complexes. 

Heterodimer formation between HBZ and cellular bZIPs has been extensively studied. For example, Thébault and colleagues showed by GST pulldown, immunoprecipitation, and immunofluorescence microscopy that HBZ and JunD colocalize and heterodimerize in vitro. In their study, HBZ and JunD heterodimers activated transcription from a luciferase construct containing the collagenase promoter [169]. Apart from a bZIP domain, HBZ also contains an amino-terminal domain (AD) with two LXXLL motifs that facilitate the recruitment of transcription cofactors. This AD, when fused to the yeast GAL4 DNA-binding domain, turns on expression from a GAL4-responsive luciferase construct and therefore has a transactivation function [159]. Interestingly, in the study Thébault and colleagues performed to assess the interaction between HBZ and JunD, the bZIP domain was required for heterodimer formation with JunD, but the AD was required to drive the HBZ/JunD-dependent activation of the luciferase reporter construct [169]. As such, the N-terminal transactivation domain provides another layer of transcriptional control to HBZ in addition to the bZIP domain. The target genes of HBZ and JunD heterodimers include viral and cellular genes. Ectopically expressed HBZ and JunD are recruited by the transcription factor specificity protein 1 (Sp1) to Sp1-biding sites on the promoter of the human telomerase reverse transcriptase (hTERT) gene in HeLa cells to turn on transcription. The HBZ- and JunD-mediated activation of the hTERT promoter requires the leucine zipper and AD of HBZ, again suggesting a synergistic relationship between the two domains [170]. HBZ and JunD, again recruited by Sp1, activate the transcription of antisense *hbz* from a recombinant HTLV-1 provirus in 293T cells to induce HBZ expression, perhaps as a feedback mechanism. In this study, HBZ expression resulted in the proliferation of HTLV-1-infected T-cell lymphoma and ATL cell lines, highlighting the importance of HBZ as a viral oncogene [171].

HBZ and CREB1 form heterodimers in vitro. In this arrangement, HBZ downregulates CREB1 binding to CREs on a luciferase-containing proviral DNA construct in 293T cells to repress viral gene expression and attenuate virion production [160]. Likewise, HBZ heterodimerizes with CREB1 to repress the transcriptional activation of the cell cycle-regulating cyclin D1 in 293T cells at a CRE site located in the cyclin D1 promoter. By this mechanism, HBZ prevents the G_1_/S transition that the viral Tax induces [163]. The repressive function HBZ exerts on CREB1 does not require the AD of HBZ [160,163].

HBZ and c-Jun colocalize in HeLa cells and form heterodimers in vitro. HBZ represses c-Jun by decreasing the association of c-Jun with TREs on target promoters and accelerating its proteasomal degradation. The HBZ-mediated repression of c-Jun transactivation also requires the AD of HBZ in addition to the leucine zipper [172].

HBZ and colleagues performed immunoprecipitation and form heterodimers in vitro. As seen with c-Jun, HBZ represses the MafB-mediated transactivation in 293T cells by enhancing the proteasomal degradation of MafB and by reducing the MafB-binding affinity for MAREs [168]. The implications of the HBZ-dependent repression of bZIPs that constitute the AP-1 complex in viral infection are not well understood but may be involved in the regulation of HTLV-1 oncogenesis, T cell proliferation, and progression to ATL [168,172].

Lastly, HBZ may also form heterodimers with the cAMP responsive element modulator (CREM) and ATF1 [160]. The peptide microarrays the Keating lab performed to study the interactions between viral and cellular bZIPs detected HBZ heterodimer formation with the members of the Jun and Maf families of bZIPs, CREB1, CREB/ATF bZIP transcription factor (CREBZF), ATF1, ATF2, and ATF7, in accordance with all studies that detected the formation of these heterodimers by GST pulldown assays, EMSA, and IPs [68]. However, the interactions between HBZ and CREM, CREBZF, ATF1, ATF2, and ATF7, and the effect of heterodimer formation on the transcriptional activation of these cellular bZIPs, have not been further studied to date. 

### 8.2. HBZ Functions That Do Not Involve Heterodimerization

The potent viral transactivator Tax binds many of the same co-activator proteins as HBZ, including CREB1 and CBP, which provides a competitive mechanism for the HBZ-mediated inhibition of Tax [159]. For example, HBZ interacts with the kinase-inducible domain interaction domain (KIX) of CBP through the LXXLL motifs in its AD to alter the binding of other transcription factors, possibly including Tax, CREB1, the proto-oncogene c-Myc [173], and forkhead box O3 (FOXO3) [166] to the CBP KIX domain. The interaction of HBZ with CBP represses the Tax-dependent transactivation from a recombinant provirus encoding the luciferase gene in 293T cells [159]. HBZ also interacts with the histone acetyltransferase (HAT) domain of CBP to inhibit the CBP-mediated acetylation of the histone H3K18, p53, and the p65 subunit of nuclear factor kappa-light-chain-enhancer of activated B cells (NF-κB) in HBZ-expressing HeLa cells. Because p53 and p65 are activated by acetylation, the HBZ-mediated inhibition of CBP histone acetyltransferase activity may prevent apoptosis and inhibit host antiviral responses [174]. Tax and HBZ also modulate the cell cycle. Tax activates NF-κB, which in turn stabilizes the mRNA of p21 and p27 to increase p21 expression and induce cell cycle arrest in Tax-expressing HeLa cells. Ectopic HBZ expression diminishes the Tax-mediated growth arrest by inhibiting NF-κB [175]. Interestingly, HBZ can also indirectly induce Tax expression. Tax expression is controlled at the post-transcriptional level by the viral protein p30^II^. The *tax* mRNA is sequestered by p30^II^ in the nucleus to reduce levels of Tax expression. The *hbz* mRNA binds the complementary *p30^II^* mRNA to potentially reduce *p30^II^* expression and increase Tax expression [176]. The HBZ suppression of Tax activity may play an important role in immune evasion by suppressing the accumulation of HTLV-1 gene products [159]. Another theory is that Tax is important for the onset of transformation, whereas HBZ is involved in the maintenance of oncogenesis [157]. The HBZ–Tax antagonism may also be regulated by the differential expression of two proteins in the different disease states of HTLV-1 infection. HBZ and Tax are only co-expressed in a small percentage of the PBMCs of HAM/TSP patients [156] and asymptomatic carriers of HTLV-1 [157].

### 8.3. HBZ Contributes to HTLV-1 Oncogenesis 

HBZ is a viral oncogene and *hbz* silencing in an ATL cell line, known as SLB-1 cells, reduces the size of tumors in mice engrafted with SLB-1 cells [177]. Transgenic mice expressing HBZ from the CD4-specific promoter spontaneously develop inflammatory lesions on the skin and in the lungs. In their study, 37.8% of transgenic mice developed T-cell lymphomas within 16 months. The mechanisms by which HBZ induces the proliferation and altered migration of T cell subsets is manifold and includes interactions with cellular transcription factors and modulations of their transcriptional activity. These cellular targets include the regulator of regulatory T cell function forkhead box P3 (FOXP3) [162], lymphoid enhancer-binding factor 1 (LEF1), T-cell-factor 1 (TCF1) of the Wnt pathway [178], signal transducer and activator of transcription (STAT) 1 and 3 [179], and ATF3 [161]. Whether HBZ and ATF3 form heterodimers and how the interaction between the two proteins leads to the proliferation of HTLV-1 infected cells remain to be elucidated. HBZ may also promote the proliferation and T cell migration at the post-transcriptional level by indirectly inducing the expression of the CC chemokine receptor 4 (CCR4) [165] and by activating host oncogenic microRNAs through unknown mechanisms [164].

HBZ also promotes viral persistence and prevents the apoptosis of HTLV-1-infected cells by interfering with the binding of the apoptosis activator FOXO3 to CBP [166] and by activating mammalian target of rapamycin (mTOR) through the inhibition of the ER stress-responsive ATF4-induced GADD34 [180]. Lastly, HBZ prevents the association of IRF1 with its target promoters in HBZ-expressing 293T cells and enhances the proteasomal degradation of IRF1 through an unknown mechanism to suppress IRF1-mediated apoptosis. The interactions between HBZ and FOXO3, GADD34, or IRF1 do not involve dimer formation and require the AD of HBZ.

Taken together, these studies demonstrate that HBZ heterodimerizes with cellular bZIPs and modulates their transactivation potential, both positively and negatively, to regulate cellular and viral gene expression. Further investigation of the functions of HBZ heterodimers could elucidate mechanisms of HTLV-1 disease progression and oncogenesis.

## 9. NS4B: A Hepatitis C Virus (HCV) bZIP Transcription Factor

HCV is a flavivirus with a single-stranded plus-sense RNA genome of about 9.6 kbp in size [181]. It is a bloodborne virus that is primarily transmitted by needle sharing, blood transfusions, and organ transplantation, but can also be transmitted through sexual intercourse [182]. HCV targets hepatocytes and enters by receptor-mediated endocytosis followed by pH-dependent membrane fusion in endocytic vesicles [181]. Unlike the viruses previously mentioned, HCV cannot establish latent infection *per se*. Instead, HCV establishes chronic infection in hepatocytes that leads to liver disease and, eventually, liver cancer. Most HCV-infected individuals are asymptomatic or display mild, often non-specific symptoms. About 25% of individuals with acute infection spontaneously clear the virus and recover, whereas 60–80% move on to develop chronic infection. Chronic infection is lifelong and progresses from hepatitis to cirrhosis in 20–30% of chronically infected individuals over the course of 20–30 years. Of those individuals that develop cirrhosis, 1–5% ultimately develop hepatocellular carcinoma [182].

### 9.1. NS4B Converts the ER into an HCV Replication Compartment

During infection, the (+)ssRNA HCV genome is directly translated into a polyprotein that is cleaved by viral and cellular proteases to yield ten individual viral proteins, one of which is the bZIP non-structural 4B (NS4B) [181,183]. NS4B is unique among the viral bZIPs because it is an integral transmembrane protein that is primarily located in the ER membrane [184,185]. NS4B is co-translationally inserted into the ER membrane [184] following cleavage of the NS4A/NS4B pre-cursor by the viral protease NS3 [186,187]. The ER retention of NS4B is mediated by two transmembrane domains in the N-terminal half of the protein [185]. NS4B induces the formation of an ER-derived intracellular structure consisting of vesicles and membranes, called the membranous web [188], where viral replication takes place [189,190,191,192]. The small guanosine triphosphate (GTP) hydrolase (GTPase) Ras-related proteins Rab-5 and Rab-7 of the endocytic pathway are cellular proteins that assist NS4B with membranous web formation by an unknown mechanism that may involve vesicle trafficking [193]. NS4B is required for viral RNA replication and virion production in HCV-infected cells [189,190,191,194,195,196]. As such, several research groups showed that mutations in the cytosolic C-terminal domain (CTD), specifically in the second of two α-helices (H2) that constitute the CTD, abrogate membranous web formation and RNA replication in adult hepatocellular carcinoma Huh-7 [190] and Huh-7.5 cells [191]. The CTD of NS4B interacts with membranes [192] but is not sufficient to cause membrane alterations [191]. For example, two lysine residues in the N-terminal amphipathic helix 1 (AH1)—immediately N-terminal to the ZIP domain—are also required for efficient viral replication and virion production and result in the altered appearance of the membranous web when mutated [196]. The requirement for NS4B in viral RNA replication is not exclusively related to the NS4B-mediated membrane alterations that are needed for the formation of viral replication complexes but may also involve the interaction of the cytosolic CTD with other viral proteins [190]. Furthermore, NS4B contains a nucleotide-binding domain that binds and hydrolyzes GTP in vitro and is required for efficient viral replication [189].

### 9.2. Structure and Function of the NS4B bZIP Domain 

NS4B contains an atypical ZIP domain at its N-terminal end in a region of the protein that forms the amphipathic helix 1 (AH1). The aa sequence of the ZIP domain varies between the NS4B isoforms expressed by the different HCV genotypes [183,196] and, like Zta, contains only a few leucine residues at position seven of the heptad repeats (Figure 2). NS4B does not contain a basic domain and has not been shown to bind DNA to date. However, 90 signal transduction-associated genes are differentially expressed in NS4B-transfected HeLa cells. These genes express cancer-related gene products, such as AP-1 and TGF-β, and also cytokine receptors, like the IL-10 receptor (IL-10R) and the IFN-γ receptor (IFNGR) [197]. How NS4B alters cellular transcription and which domain of the protein might mediate transcriptional regulation remains to be elucidated.

**Figure 5:**
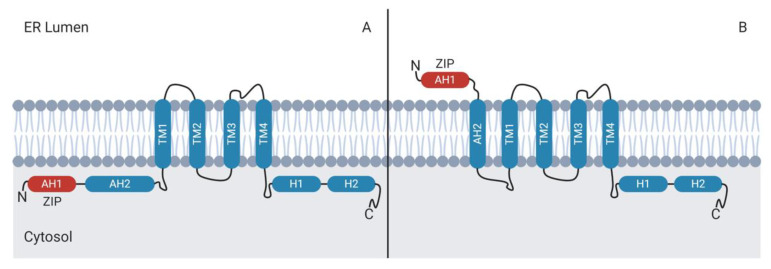
Topology of NS4B in the ER membrane. (**A**) The AH2 amphipathic helix domain can be positioned on the cytosolic face of the ER, along with the amino-terminal AH1 domain. (**B**) Insertion of AH2 in the ER membrane translocates the AH1 domain to the ER lumen. It is not yet known how altered NS4B topology affects protein function. TM: transmembrane, AH: amphipathic helix.

NS4B displays a dual membrane topology (Figure 5). Following the NS3-dependent cleavage of NS4B from NS4A in the cytoplasm, the N-terminal ZIP domain α-helix (AH1) and the adjacent amphipathic helix (AH2) protrude into the cytoplasm. The two helices can cross the ER membrane such that the ZIP domain protrudes into the lumenal side of the ER membrane and the adjacent amphipathic helix forms a fifth transmembrane domain [186,187,196]. The processes that mediate the translocation of the NS4B N-terminus across the ER membrane are poorly understood, but may involve the oligomerization of AH2 [196].

The role of the ZIP domain in the various functions of NS4B has not been well studied to date. The leucine zipper may be required for efficient viral replication. For instance, aa substitutions of the “a” and “d” position residues in the heptad repeats reduce viral replication in the luciferase-encoding Huh-7-Lunet cell line [198]. The replication-associated protein–protein interactions mediated by the ZIP domain during viral replication remain unknown. NS4B can form homodimers [183], but it is unknown how homodimer formation affects NS4B function. 

### 9.3. NS4B and ER Stress

**Figure 6:**
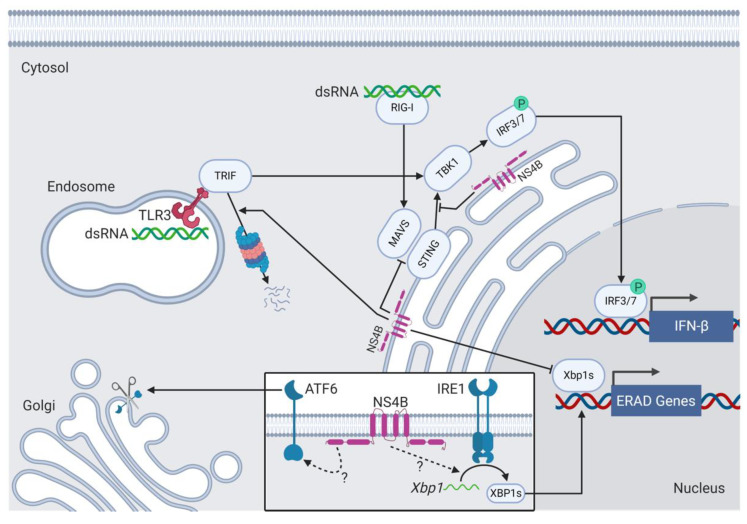
NS4B inhibits innate immune signal transduction and interacts with unfolded protein response sensor proteins. Cytoplasmic RIG-I and endosomal TLR3 sense HCV dsRNA and signal through MAVS and TRIF, respectively, leading to the TBK1-dependent phosphorylation and the activation of IRFs that drive IFN-β expression in the nucleus. The viral bZIP and transmembrane protein NS4B prevent the association of MAVS with STING and lead to the enhanced proteasomal degradation of TRIF to inhibit IFN-β production. NS4B also induces ATF6 cleavage and Xbp1 splicing by unknown mechanisms and therefore induces the UPR. NS4B inhibits the XBP1s-dependent expression of ER-associated degradation genes.

NS4B interacts with the ER-resident stress sensor ATF6β [199]. ATF6β, like its homologue ATF6α, is activated during ER stress and cleaved in the Golgi to yield ATF6β(N). ATF6α and ATF6β share high aa sequence homology in all regions except for their N-terminal transactivation domains (TADs). Because of the differences between their respective TADs, ATF6α and ATF6β carry out different functions. As such, ATF6β(N) is an antagonist of ATF6α(N) and represses its ability to activate ATF6-responsive ER stress-associated genes, perhaps to fine-tune the UPR [200]. NS4B and ATF6β interact in a yeast two-hybrid screen and co-localization in HeLa cells. The association of the two proteins requires the bZIP domain of ATF6β and the N-terminal domain of NS4B [199] (Figure 6). Because the N-terminus of NS4B contains the atypical ZIP domain, NS4B and ATF6β may associate as heterodimers. When embedded in the ER membrane, the bZIP domain of ATF6 protrudes into the cytoplasm [201]. Thus, the interaction between the ZIP domains of the membrane-bound proteins NS4B and ATF6β may occur on the cytoplasmic side of the ER membrane. However, heterodimer formation between NS4B and ATF6β has not been further investigated to date and the implications of the NS4B–ATF6β interaction are unknown. Furthermore, NS4B binding to ATF6α remains to be investigated.

NS4B activates the UPR (Figure 6). The ectopic expression of NS4B causes ATF6 cleavage and IRE1-dependent *Xbp1* mRNA splicing in hepatocellular carcinoma Hep3B and Huh-7 cell lines treated with tunicamycin [202]. Tunicamycin is an antibiotic that prevents N-linked glycosylation and results in protein misfolding, which induces ER stress. Although NS4B expression in Huh-7 and HeLa cells results in *Xbp1* splicing, XBP1s protein fails to transactivate the downstream target gene ER degradation-enhancing alpha-mannosidase-like protein 1 (EDEM1), the protein product of which is involved in ERAD [203]. As such, NS4B may selectively activate the UPR, but suppress transcription of downstream target genes with antiviral functions. The mechanisms by which NS4B activates ATF6 cleavage and *Xbp1* splicing are unknown.

UPR activation and modulation is not unique to HCV. Many viruses trigger ER stress, at least in part by overloading the ER protein folding machinery with a burst of secreted and transmembrane viral proteins during viral infection. The UPR aims to decrease the protein load by attenuating translation, increasing protein turnover, and initiating apoptosis if stress persists. For these reasons, the UPR can take on an antiviral role during viral infection. Some viruses have therefore evolved to modulate the UPR to optimize viral replication. Lytic KSHV, for example, activates all three sensors of the UPR to facilitate efficient viral replication but blocks the transcriptional responses downstream of receptor activation that would otherwise restrict viral replication [204]. Lytic MDV activates the ATF6 and IRE1 arms of the UPR but suppresses the PERK arm to prevent apoptosis of MDV-infected cells [205]. Interestingly, XBP1s can stimulate reactivation from latency for both KSHV [206] and EBV [207]. Little is known about the effect of HCV-mediated UPR activation on viral fitness and survival, but it can be assumed that UPR activation confers some benefit to the viral life cycle. For example, the induction of the UPR and autophagy is beneficial for HCV replication. The treatment of HCV-infected adult hepatocellular carcinoma (OR6) cells with UPR-inhibiting drugs reduces HCV replication [208]. ATF6α(N) induces the expression of chaperones, which could assist in the folding of viral proteins. Likewise, the NS4B-mediated inhibition of the XBP1s-dependent expression of ERAD genes, like EDEM1, may promote viral protein production by preventing the ERAD-mediated degradation of ER-localized HCV proteins [203].

### 9.4. The Role of NS4B in Oncogenesis and Immune Evasion

NS4B is an oncogene that contributes to HCV oncogenesis and hepatocellular carcinoma. For example, early studies showed that ectopically expressed NS4B and the cellular oncogene Harvey rat sarcoma (H-Ras) co-operatively lead to the transformation of NIH 3T3 mouse embryonic fibroblasts [209]. In human hepatocytes, NS4B activates the ER overload response (EOR), which is a cellular ER stress-responsive pathway that triggers Ca2+ mobilization and induces the production of reactive oxygen species (ROS) in the mitochondria [202]. The NS4B-dependent activation of the EOR also results in the activation of NF-κB [202] and the cancer-related STAT3 pathway to induce cell transformation [210]. To further promote cell proliferation, NS4B, through its cytoplasmic C-terminal domain, interacts with the cellular tumor suppressor and membrane protein Scribble and mediates the degradation of Scribble in a proteasome-dependent manner by an unknown mechanism [211].

The mechanisms by which NS4B facilitates viral immune evasion are well documented (Figure 6). For example, NS4B inhibits IFN-β production. The cytoplasmic retinoic acid-inducible gene-I (RIG-I) senses viral double-stranded RNA and activates mitochondrial antiviral-signaling protein (MAVS). MAVS then activates the ER and mitochondrial transmembrane protein STING, which associates with TBK1 to phosphorylate and activate IRF3 and IRF7, which then turn on the expression of IFN genes [212]. NS4B associates with STING through its C-terminal domain and blocks MAVS from interacting with STING [213], thereby preventing STING activation, accumulation, and downstream signaling [214]. NS4B also disrupts interactions between STING and TBK1 [215]. NS4B can also inhibit TLR3-dependent IFN-β production. Like RIG-I, the endosomal TLR3 senses double-stranded RNA and signals the downstream production of IFN-β. NS4B mediates the proteasome-dependent degradation of the TLR3 adaptor TIR-domain-containing adapter-inducing interferon-β (TRIF) by an unknown mechanism and thereby inhibits IFN production [216]. Other functions that NS4B mediates in co-operation with additional viral proteins, like NS4A, and cellular proteins include lipogenesis [217], the induction of autophagy [218] and apoptosis [219], and the attenuation of host translation and protein production (host shutoff) [220,221].

In summary, NS4B remains a poorly understood and structurally atypical bZIP protein that primarily functions as a membrane-altering transmembrane protein that is indispensable for HCV replication. NS4B provides the structural framework for the formation of viral replication compartments and extensively modulates the functions of players of the type 1 IFN pathway to facilitate viral immune evasion and persistence. NS4B resides in close proximity to ER-stress sensors and activates the UPR. Further research could provide insight into the functional role of the NS4B ZIP domain in UPR activation and other biological outcomes.

## 10. Conclusions

Through co-evolution with their hosts, many viruses have acquired host genes that encode gene products that commandeer host cell functions to ensure efficient viral replication and spread to new hosts [52]. Viral bZIPs have been identified in four different human viruses and one avian virus to date. Three of the five bZIP-encoding viruses, EBV, KSHV, and MDV, are herpesviruses with large dsDNA genomes, whereas the retrovirus HTLV-1 and the flavivirus HCV have small ssRNA genomes. Among these viruses, only EBV and KSHV share significant genetic similarly. The divergence of these viruses suggests that viral ancestors acquired host bZIP genes through independent events. The five viruses differ in their host range, tissue tropism, and viral life cycle, but have in common the ability to regulate cell proliferation and oncogenesis. Although MEQ, HBZ, and NS4B are oncoproteins that drive proliferation by different mechanisms, Zta and K-bZIP primarily act as transactivators of the viral lytic cycle and inhibitors of cell proliferation. Coincidentally, Zta and K-bZIP are expressed by herpesviruses with large genomes; for these viruses, oncogenesis results from the coordinated action of multiple viral gene products, which likely relegates the viral bZIP proteins to an auxiliary role. 

Most viral bZIP proteins interact with transcription factors to influence viral and host gene expression. Immune evasion and cell cycle control are also common features of these proteins. Except for NS4B, which lacks a basic domain, all viral bZIPs have been demonstrated to bind DNA as homodimers and/or heterodimers. Dimerization affects the functions and DNA-binding specificities of cellular bZIPs and viral bZIPs alike. As such, MEQ homodimers recognize a unique 5′-RACACACAY-3′ motif [143] and facilitate repressive functions [141]. By contrast, MEQ and Jun heterodimers preferentially bind TREs and CREs and function as transcriptional activators [141]. HBZ exclusively heterodimerizes with cellular bZIPs and selectively activates [169,170] or inhibits [160,163,167,172] cellular and viral transcription. The lack of homodimer formation is not unique to viral bZIPs, because Fos likewise only forms heterodimers [68]. By contrast, Zta and K-bZIP do not form heterodimers, but may associate with C/EBPα as higher-order oligomers [86,94]. The ZIP and basic domains of viral bZIPs adhere to the sequence consensus less tightly than cellular bZIPs (Figure 2). K-bZIP and HBZ contain atypical basic domains, whereas Zta and NS4B contain atypical ZIP domains. Regardless of these structural abnormalities, most viral bZIPs function as DNA-binding transcription factors. Viral bZIPs recognize unique DNA-binding motifs [101,142], as well as classical bZIP DNA motifs, such as CREs [142,163], TREs [67,142], MAREs [168], and CAAT boxes [97], as homodimers and/or heterodimers. Unlike cellular bZIPs, viral bZIPs have evolved to carry out a greater variety of functions that pertain to viral replication and fitness. Not all functions these bZIPs carry out involve their bZIP domains. Indeed, K-bZIP has SUMO E3 ligase activity [116], MEQ contains an N-terminal proline-rich transactivation domain apart from its bZIP domain [142], HBZ has an LXXLL-containing N-terminal transactivation domain [159], and NS4B is primarily a transmembrane protein that re-shapes cellular membranes to allow viral replication compartments to form [184,188]. We speculate that viral bZIPs may have acquired additional properties not normally associated with host bZIP proteins. Alternatively, the discovery of non-canonical properties of viral bZIP proteins may indicate that host bZIP proteins also have non-canonical properties worthy of future investigation. As we have learned, viruses are excellent teachers, and the study of viral proteins frequently advances our understanding of the fundamental regulation of many cellular processes. Thus, the viral bZIP proteins may still have more to teach us about the inner workings of the cell.
